# Ideal suturing technique for robot-assisted microsurgical anastomoses

**DOI:** 10.1007/s11701-024-02012-7

**Published:** 2024-06-29

**Authors:** Kai J. Wessel, Isa Wendenburg, Charalampos Varnava, Sascha Wellenbrock, Alexander Dermietzel, Mirkka Hiort, David Kampshoff, Philipp Wiebringhaus, Tobias Hirsch, Maximilian Kueckelhaus

**Affiliations:** 1 Department of Plastic Surgery, University Hospital Muenster, Albert-Schweitzer-Campus 1, 48157 Muenster, Germany; 2 Department of Plastic and Reconstructive Surgery, Institute of Musculoskeletal Medicine, University of Muenster, Muenster, Germany; 3 Department of Plastic, Reconstructive and Aesthetic Surgery, Hand Surgery, Fachklinik Hornheide, Muenster, Germany

**Keywords:** Robotics, Microsurgery, Symani, RoboticScope, Anastomosis, Suture, Knot

## Abstract

The development of novel robotic devices specifically designed for open microsurgery leads to increasing applications in reconstructive procedures. While initial studies revealed improved precision and surgical ergonomics upon robotic assistance, surgical time was consistently observed to be increased. This study compares two robotic suturing techniques using the Symani Surgical System and RoboticScope in a preclinical setting, to further leverage the benefits of novel robotic devices in microsurgery. Six experienced microsurgeons performed three microvascular anastomoses with a “steady-thread” suturing technique and a “switch-thread” technique on 1.0-mm-diameter artificial silicone vessels. Time for anastomosis and participant’s satisfaction with the techniques and robotic setup were recorded. Anastomosis quality and microsurgical skills were assessed using the Anastomosis Lapse Index and Structured Assessment of Microsurgery Skills. Lastly, technical error messages and thread ruptures were quantified. Knot tying was significantly faster and evaluated significantly better by participants using the steady-thread technique (4.11 ± 0.85 vs. 6.40 ± 1.83 min per anastomosis). Moreover, microsurgical skills were rated significantly better using this technique, while both techniques consistently led to high levels of anastomosis quality (2.61 ± 1.21 vs. 3.0 ± 1.29 errors per anastomosis). In contrast, the switch-thread technique was associated with more technical error messages in total (14 vs. 12) and twice as many unintended thread ruptures per anastomosis (1.0 ± 0.88 vs. 0.5 ± 0.69). This study provides evidence for the enhanced performance of a steady-thread suturing technique, which is suggested to be applied upon robot-assisted microsurgical procedures for optimized efficiency.

**Supplementary Information:**

The online version contains supplementary material available at 10.1007/s11701-024-02012-7.

## Introduction

Robot-assisted microsurgery in plastic surgery has become increasingly popular due to its potential to improve accuracy, safety and surgical ergonomics of procedures. Novel robotic systems are equipped with specialized tools and instruments that enable the surgeon to perform difficult tasks with greater precision and accuracy compared to traditional techniques. The key features of such systems are motion scaling and elimination of tremors, allowing for ultimate control over the instruments when handling (sub)-millimeter structures. The only currently available system specifically designed for open microsurgery is the Symani Surgical System (Medical Microinstruments Inc., Wilmington, DE, USA). It offers wristed microsurgical and supermicrosurgical instruments, adding distal motion axes for an improved range of motion compared to conventional microsurgical instruments. Several preclinical studies revealed improved precision and ergonomics upon application of the Symani for the performance of microvascular anastomoses in vitro [1–3] and in vivo [4]. Furthermore, feasibility and safety of robot-assisted microsurgery was demonstrated in multiple initial clinical trials. Therefore, successful application of the Symani has been described in the fields of lymphatic surgery [5–7], extremity reconstruction [8–10], autologous breast reconstruction [11, 12] and peripheral nerve surgery [13, 14].

However, in spite of steep learning curves upon introduction of novel robotic systems [2, 15], most studies consistently revealed increased surgical times of robot-assisted procedures and anastomoses compared to conventional approaches [5, 10, 11, 16]. In order to fully leverage the benefits of robotic technology and to guarantee the best possible results of microvascular anastomoses, we sought to determine a robotic suturing technique combining time efficiency, precision and accuracy on a high level. Here, we describe two suturing techniques for robot-assisted microsurgical anastomoses and compare their speed, quality and error susceptibility in a preclinical setting, using the Symani Surgical System in combination with the RoboticScope (BHS Technologies, Innsbruck, Austria) for the performance of microvascular anastomoses on artificial silicone vessels.

## Materials and methods

### Setup

Microsurgical anastomoses were performed at our clinic’s microsurgery laboratory. The Symani was utilized for robot-assisted microsurgical anastomoses. This system provides wristed microsurgical and supermicrosurgical instruments, with motion scaling from 7 to 20 ×, tremor filtration, and increased range of motion through additional distal motion axes. The surgeon sits in a highly ergonomic chair and operates the system using wired controllers that resemble forceps, which can be freely moved and rotated while in an ergonomic position. All movements are transmitted with high precision and in real time to two robotic slave arms. The operating unit can be covered with sterile drapes and positioned above the desired operating field with flexibility.

The robotic digital microscope RoboticScope is a high-definition camera system that is connected to an augmented reality headset. It projects a high-quality, stereotactic image in front of the surgeon’s eyes, creating a three-dimensional live image. The surgeon’s head movements are converted onto the system through motion tracking using a multi-axis robotic arm. The surgeon can navigate through an augmented menu that appears on top of the operating field image using head gestures. This allows for the adjustment of zoom and focus, changes in orbital view, navigation through the operating field, and image/video recording completely freehand, without interrupting the surgery. The RoboticScope is equipped with a high-resolution camera, which was used for all video and photo recordings.

Participants were seated away from the operation table to perform the anastomoses, thereby being able to maintain an optimal ergonomic position.

### Study population

Six experienced microsurgeons from our institution with more than 5 years of experience in free flap reconstruction participated in this study. Participants previously underwent a comprehensive training on operating the robotic systems and performed multiple anastomoses with the investigated approach until reaching a steady state in the preclinical learning curve. Each study attendee completed six robot-assisted microvascular end-to-end anastomoses on 1.0-mm-diameter artificial silicone vessels (WetLab, Japan) with six stitches of 10-0 sutures (Ethilon, Ethicon, USA), three on the frontside and three on the backside after flipping the vessel. Three anastomoses were performed with each suturing technique, respectively (see below). Silicone vessels were stabilized using a microvascular approximator on a foam training platform.

### Suturing techniques

**Fig. 1:**
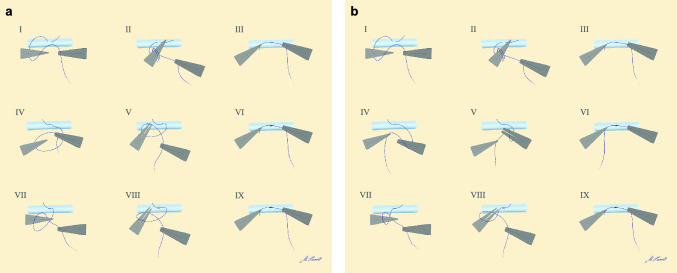
Suturing techniques. Illustration of the essential steps of **a** the steady-thread suturing technique and **b** the switch-thread suturing technique. A detailed description of both techniques can be found in “Materials and methods”. Moreover, the techniques are demonstrated in Supplementary video 1 and 2

The steady-thread suturing technique (steady technique) and the switch-thread suturing technique (switch technique) are illustrated in Fig. 1 and Supplementary Video 1 and 2 for better understanding. The robotic micro dilator is operated with the non-dominant hand (in this study always the left hand), while the robotic micro needle holder with inbuilt scissors close to the joint of the instrument is operated with the dominant hand (in this study always the right hand).

The steady technique describes the suturing technique, which is mainly used for conventional microanastomoses at our institution, and was adapted to the robotic approach. The long end of the thread is held with the needle holder and double-looped around the dilator. Then, the short end of the thread is grasped with the dilator and pulled through the loop. The long end of the thread is kept with the needle holder for the second and third knot. It is now single-looped around the dilator and the short end is pulled through again. Finally, it is single looped around the dilator in the opposite direction, the short end is pulled through and the thread is cut with the inbuilt scissors (Fig. 1a, Sup. Vid. 1).

The switch technique describes the suturing technique, which was proposed by the manufacturer for achieving square knots through robot-assisted suturing. The first knot is performed in the same manner as with the steady technique. However, then the long end of the thread is passed from the needle holder to the dilator and single-looped around the needle holder, which pulls the short end of the thread through the loop in the opposite direction. For the third knot, the long end is passed to the needle holder again and single-looped around the dilator, which grasps and pulls the short end. Finally, the thread is cut with the inbuilt scissors (Fig. 1b, Sup. Vid. 2).

In short, the main difference between the two techniques is whether the thread is kept with the same instrument for each knot to save steps and time (steady technique), or if it is passed between the instruments after each knot to prevent crossing and collision of the robotic instruments (switch technique).

### Data collection and processing

During each microvascular anastomosis, the time to complete the anastomosis was recorded. Using video recordings, the total time per anastomosis was divided into the major steps of each anastomosis, which were analyzed separately: needle positioning, piercing, passage through vessel wall, knot tying, cutting of suture and additional time.

After finishing each anastomosis, participants had to fill out a questionnaire evaluating their subjective satisfaction with the anastomosis and the knot technique, as well as their satisfaction with the Symani performance, RoboticScope performance, and combined performance of both systems on a numeric rating scale from 0 to 10 (0 = minimum, 10 = maximum).

To assess the quality of microvascular anastomoses, the Anastomosis Lapse Index (ALI) was applied. This involved cutting the anastomoses longitudinally and photographing the inside. Deidentified and blinded photographs were analyzed by a single reviewer to identify specific types of errors and the total number of errors, that were previously described by Ghanem et al*.* (anastomosis line disruption, backwall or sidewall catch, oblique stitch causing distortion, bite leading to tissue infoldment, partial thickness stitch, unequal distancing of sutures, visible tear in vessel wall, strangulation of tissue edges, thread in lumen, large edge overlap) [17].

Furthermore, microsurgical skills using the different suturing techniques were analyzed by videorecording the procedures and evaluating the deidentified and blinded videos. An experienced microsurgeon used a modified version of the Structured Assessment of Microsurgery Skills (SAMS) by van Mulken et al. [18] to assess all anastomoses. The modified SAMS evaluates dexterity (steadiness, instrument handling, tissue handling), visuo-spatial ability (suture placement, knot technique) and operative flow (steps, motion, speed), as well as the overall performance and indicative skill level on a numeric rating scale from 1 to 5 (5 representing excellent skills).

Lastly, technical error messages generated by the Symani during anastomoses interrupting the workflow were recorded and quantified. Possible error messages included: master moved too quickly, device exceeded motion range, joint of device at pivot stop, master outside console workspace and device at workspace boundary. In addition, the number of threads torn unintentionally with both suturing techniques was counted and the total number of threads used per anastomosis was documented (only if the thread was too short after rupture, a new thread was used).

### Statistical analysis

Statistical analysis was performed using GraphPad Prism (GraphPad Software Inc., USA). In all plots and bar charts, dots represent individual values with arithmetic mean and standard deviation. Statistical significance was assessed for surgical time, questionnaire items, ALI scores, SAMS scores and thread count using a two-way ANOVA when comparing multiple groups (corrected for multiple comparisons with Tukey and Sidak test, 95% confidence interval) and Student’s *t*-test when comparing the means of two groups (unpaired, two-tailed, 95% confidence interval). *P* values < 0.05 were considered statistically significant.

## Results

### Assessment of surgical time and subjective satisfaction

**Fig. 2:**
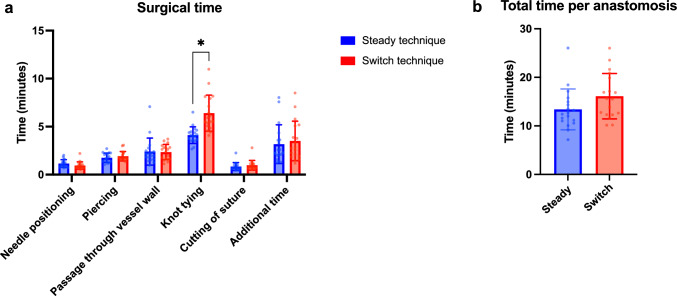
Surgical time. The time taken for each anastomosis was recorded and divided into different steps: needle positioning, piercing, passage through vessel wall, knot tying, cutting of suture and additional time. **a** The time required for each of these separate steps per anastomosis and **b** the total time per anastomosis from the initial needle positioning to the last suture cutting were quantified. Bar charts display mean values with standard deviation, dots represent individual values (*n* = 18, *p < 0.05)

The surgical time required to complete each anastomosis was divided into major steps, which were analyzed separately. On average per anastomosis, needle positioning took 1.16 ± 0.39 min with the steady technique and 0.95 ± 0.37 min with the switch technique, piercing took 1.76 ± 0.47 min with the steady technique and 1.92 ± 0.48 min with the switch technique, passage through vessel wall took 2.40 ± 1.36 min with the steady technique and 2.35 ± 0.77 min with the switch technique, knot tying took 4.11 ± 0.85 min with the steady technique and 6.40 ± 1.83 min with the switch technique, cutting of suture took 0.85 ± 0.40 min with the steady technique and 0.98 ± 0.49 min with the switch technique and the additional time was 3.17 ± 1.96 min with steady technique and 3.51 ± 2.00 min with the switch technique (Fig. 2a). Thereby, most steps did not significantly differ between the two approaches. However, knot tying, which distinguishes the two techniques, was significantly faster with the steady technique (*p* = 0.000043). The total time per anastomosis from the initial needle positioning to the last suture cutting was 13.40 ± 4.06 min with the steady technique and 16.10 ± 4.11 min with the switch technique (Fig. 2b). Thereby, the steady technique was faster overall, however, not statistically significant (*p* = 0.0769).

**Fig. 3:**
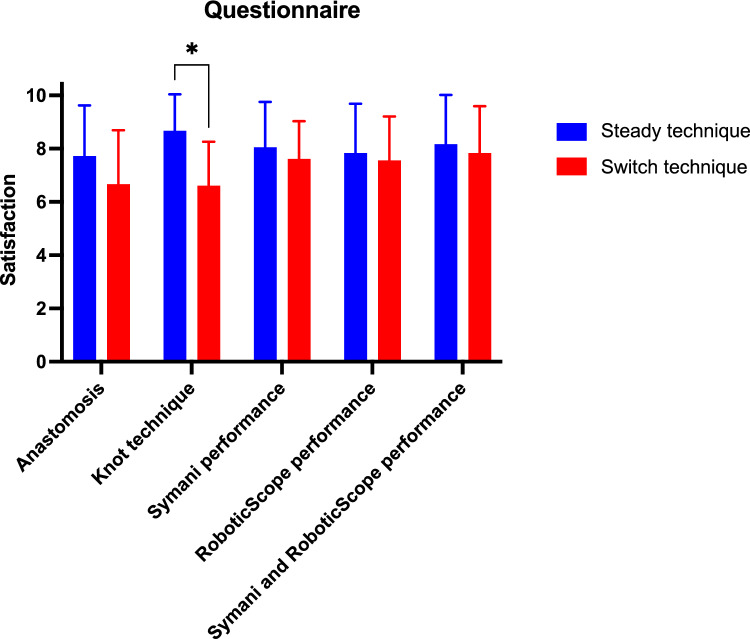
Questionnaire. Participants filled out a questionnaire after each anastomosis, evaluating their satisfaction with the anastomosis, knot technique, Symani performance, RoboticScope performance and the combined performance of both systems on a numeric rating scale from 0 (minimum) to 10 (maximum), distinguishing by the applied suturing technique. Bar chart represents mean evaluations with standard deviation (*n* = 18, *p < 0.05)

After completion of each anastomosis, participants evaluated different aspects on a questionnaire (steady vs. switch technique). Subjective satisfaction with the anastomoses in general was evaluated better with the steady technique (7.72 ± 1.85 points vs. 6.67 ± 1.97 points), while especially the knot technique was evaluated significantly better with the steady technique (8.67 ± 1.33 points vs. 6.61 ± 1.60 points, *p* = 0.000269). Symani performance, RoboticScope performance and the combined performance of both systems also showed slightly better evaluations with the steady technique; however, they were consistently on a high level with both techniques, never dropping below 7.72 out of 10 points (Fig. 3). Thus, the steady technique was overall preferred by participants, mostly attributed to the knot technique.

### Anastomosis quality and microsurgical skills

**Fig. 4:**
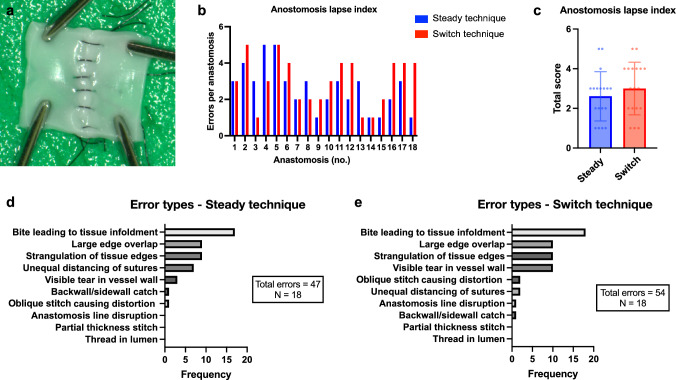
Anastomosis lapse index (ALI). All anastomoses were cut longitudinally and everted to assess the ALI, quantifying anastomosis quality (*n* = 18). **a** Representative luminal image of an anastomosis prepared for ALI assessment. **b** Total errors of each consecutive anastomosis were counted for both techniques and **c** mean errors per anastomosis were calculated with standard deviation (dots represent individual values). The total number of each distinct error type using **d** the steady-thread technique and **e** switch-thread technique was further quantified

Anastomosis quality was assessed using the ALI score (Fig. 4a) and total errors per anastomosis were determined for each anastomosis with both techniques (Fig. 4b). On average 2.61 ± 1.21 errors per anastomosis occurred when using the steady technique compared to 3.0 ± 1.29 errors per anastomosis when using the switch technique (Fig. 4c).

**Fig. 5:**
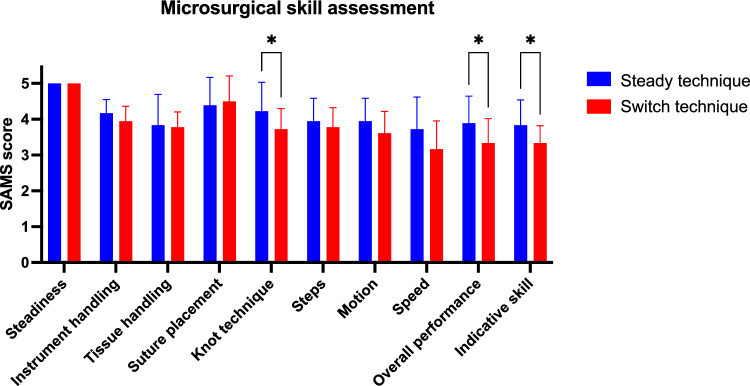
Structured assessment of microsurgery skills (SAMS). The performance of anastomoses was videorecorded and microsurgical skills were evaluated in a blinded fashion by an experienced microsurgeon according to a modified version of the SAMS score. Bar chart represents mean scores of individual categories and summative assessments from 1 (worst) to 5 (best) with standard deviation (*n* = 18, *p < 0.05)

Furthermore, microsurgical skills were assessed by an experienced microsurgeon according to a modified version of the SAMS score. Most SAMS categories, such as “steadiness”, “instrument handling”, “tissue handling”, “suture placement”, “steps” and “motion” were consistently evaluated at proficient levels with both techniques, ranging between 3.5 and 5.0 points. However, “knot technique” was evaluated significantly better with the steady technique (*p* = 0.039) and also “speed” showed slightly non-significantly better evaluations with this technique (*p* = 0.056), which resulted in a significantly improved “overall performance” (*p* = 0.027) and “indicative skill” (*p* = 0.019), when using the steady technique for microsurgical anastomoses (Fig. 5). Thereby, anastomosis quality and microsurgical skills were consistently evaluated on high levels with both techniques; however, the steady technique performed slightly better on both scores.

### Error messages and thread count

**Fig. 6:**
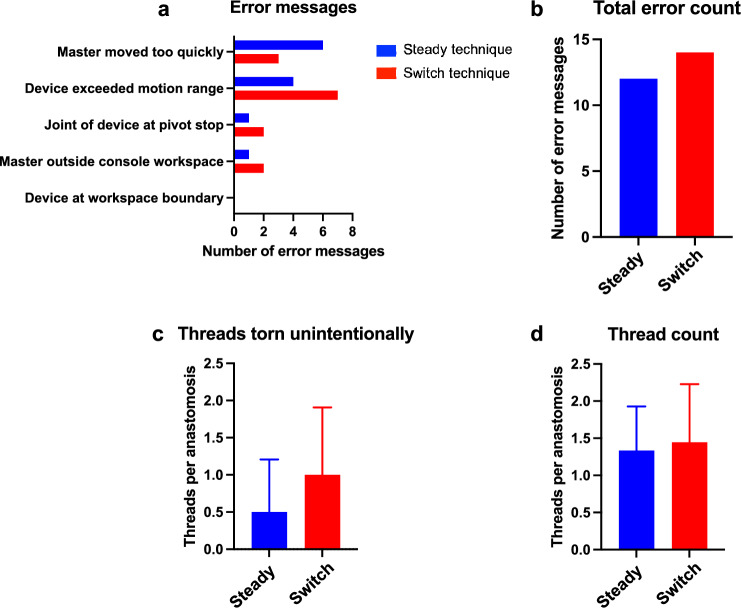
Error messages and thread count. **a** Distinct error messages generated by the Symani during the performance of anastomoses interrupting the workflow were counted for both techniques and **b** the total number of error messages per technique was calculated. Moreover, **c** the mean number of threads torn unintentionally per anastomosis and **d** the mean number of threads used per anastomosis were quantified for each technique with standard deviation (*n* = 18)

Technical error messages generated by the Symani interrupting the workflow were quantified for both techniques. Regarding specific error messages, “device exceeded motion range”, “joint of device at pivot stop” and “master outside console workspace” occurred more often with the switch technique, while “master moved too quickly” occurred twice as often with the steady technique and “device at workspace boundary” did not occur at all with both techniques (Fig. 6a). Overall, 12 error messages were generated during 18 anastomoses using the steady technique and 14 error messages using the switch technique (Fig. 6b).

Moreover, the number of threads torn unintentionally per anastomosis and the total number of threads used per anastomosis were recorded. Notably, the switch technique was associated with twice as many thread ruptures per anastomosis compared to the steady technique (steady: 0.5 ± 0.69 vs. switch: 1.0 ± 0.88) (Fig. 6c). Nevertheless, the total number of threads used per anastomosis was comparable between both techniques (steady: 1.3 ± 0.59 vs. switch: 1.4 ± 0.78), since most threads were still long enough to be reused after rupture (Fig. 6d).

Altogether, performance of microanastomoses was still more efficient with the steady technique than with the switch technique, regarding workflow interruptions by technical error messages and thread ruptures.

## Discussion

In recent years, major advancements in the development robotic surgical assistance devices and robotic surgical microscopes have led to the admission of novel robotic systems specifically designed for open microsurgery into clinical practice. Thereby, novel areas of robot-assisted procedures are gradually being investigated especially in the field of plastic surgery and microsurgery. The Symani system has already been successfully applied for lymphatic surgery [5–7], reconstructive free flap surgery [8–12] and peripheral nerve surgery [13, 14], consistently revealing improved surgical ergonomics and microsurgical precision compared to conventional manual approaches. Importantly, during these procedures, in its current configuration the Symani is only being used for the performance of microsurgical anastomoses, while the whole preparation is performed conventionally. Nevertheless, at the current state of knowledge, surgical time appears to be a specific drawback of robotic procedures, as it was shown to be increased in most studies [5, 10, 16]. To further improve time efficiency, we sought to determine an ideal suturing technique for robot-assisted microsurgical anastomoses without impairing anastomosis quality.

Upon preclinical training with the Symani system, a suturing technique that involves switching the thread ends between instruments after each knot was suggested by the manufacturer (switch-thread technique), as it prevents collision of the robotic instruments, requires less rotation and bending of instrument tips and leads to more intuitive folding of each square knot in the correct orientation, thereby being easier to apply upon training with the novel system. However, the suturing technique applied for microanastomoses during conventional manual microsurgery at our institution is performed in a different manner, keeping the long thread end with the needle holder for all three knots and opposing the orientation the thread is looped around the dilator each time, in order to tighten the knots correctly (steady-thread technique).

Results from this study revealed that knot tying with the steady technique is indeed significantly faster compared to the switch technique, also leading to an overall improvement of anastomosis time. Consistently, the steady technique was evaluated significantly better by experienced microsurgeons on a numeric rating scale, with high levels of satisfaction with the robotic setup in general using both approaches. Importantly, the quality of microanastomoses assessed by the ALI score was shown to be on comparably proficient levels with both techniques, not showing statistically significant differences. Thereby, it is demonstrated that the improvement of surgical time was not met with an impairment of anastomosis quality. On the contrary, microsurgical skills assessed by the SAMS score were even improved using the steady technique, mostly attributed to significant improvements in the category “knot technique”.

On the other hand, workflow interruptions by technical error messages generated by the Symani system occurred more often with the switch technique. These error messages appear for example, when the threshold of the workspace is reached with one of the masters, when the master is moved too quickly, or when the motion range of the robotic instruments is exceeded either regarding the rotation and bending limits or the motion range of the robotic arms. Upon such an event, a quick resynchronization of the instruments is required, leading to a short delay of the procedure, which should, therefore, be avoided.

Furthermore, workflow interruptions by thread ruptures occurred twice as often with the switch technique compared to the steady technique, potentially requiring the usage of a new thread, which causes another delay in surgical time and elevated costs for suture material. Interestingly, it was observed that thread ruptures were mostly not caused by too intense force application during tightening the knots or accidentally cutting the thread with the inbuilt scissors in the needle holder, but repetitively by sharp edges and corners of the instruments upon looping the thread around the needle holder, where it was prone to entanglement. Since only the needle holder has these edges, while the dilator is very smooth, this provides another advantage for the steady technique, where the thread is kept with the needle holder and only looped around the dilator in different orientations.

Summarizing, it was demonstrated that suturing with the steady-thread technique is superior over the switch-thread technique considering time efficiency, microsurgical skills and error susceptibility, without affecting the quality of microanastomoses. Therefore, we suggest the steady-thread technique for robot-assisted microsurgical procedures, providing an approach to improve the time of robotic microanastomoses. Nevertheless, since the vascular or nerval anastomosis only accounts for a small portion of the overall surgical time, further research investigating additional concepts to further optimize robot-assisted microsurgical procedures is needed in order to fully leverage the benefits of novel robotic systems.

## Supplementary Information

Below is the link to the electronic supplementary material.

Supplementary file1 (MOV 59697 KB)

Supplementary file2 (MOV 87858 KB)

## Data Availability

The data that support the findings of this study are available from the corresponding author, MK, upon reasonable request.
